# A Dual-Function Wearable Electrochemical Sensor for Uric Acid and Glucose Sensing in Sweat

**DOI:** 10.3390/bios13010105

**Published:** 2023-01-06

**Authors:** Zhanhong Li, Yuwei Wang, Zheyuan Fan, Yufan Sun, Yue Sun, Yiduo Yang, Yifan Zhang, Junjie Ma, Zifeng Wang, Zhigang Zhu

**Affiliations:** School of Health Sciences and Engineering, University of Shanghai for Science and Technology, Shanghai 201209, China

**Keywords:** uric acid, glucose, wearable, sensor, sweat

## Abstract

Simultaneous detection of uric acid and glucose using a non-invasive approach can be a promising strategy for related diseases, e.g., diabetes, gout, kidney disease, and cardiovascular disease. In this study, we have proposed a dual-function wearable electrochemical sensor for uric acid and glucose detection in sweat. The sensor with a four-electrode system was prepared by printing the ink on a common rubber glove. CV and chronoamperometry were used to characterize the prepared sensor’s electrochemical sensing performance. The sensors exhibited the linear range from 0 to 1.6 mM and 0 to 3.7 mM towards uric acid and glucose electrochemical sensing in phosphate-buffered solution, with the corresponding limit of detection of 3.58 μM and 9.10 μM obtained, respectively. Moreover, the sensors had shown their feasibility of real sample sensing in sweat. The linear detection range for uric acid (0 to 40 μM) and glucose (0 to 1.6 mM) in the sweat can well cover their concentration range in physiological conditions. The prepared dual-function wearable electrochemical sensor features easy preparation, fast detection, high sensitivity, high selectivity, and the practical application potential in uric acid and glucose sensing.

## 1. Introduction

Monitoring the concentration of molecules related to physiological metabolism in biofluid (such as blood, sweat, saliva, urine, tears, and interstitial fluid) is a widely adopted strategy for health management and disease diagnosis and treatment. E.g., the blood glucose level is an essential criterion for diabetes diagnosis. According to the report of World Health Organization (WHO) [1], the number of people with diabetes rose from 108 million in 1980 to 422 million in 2014, and there are more than 95% of people with diabetes have type 2 diabetes. Moreover, between 2000 and 2019, there was a 3% increase in diabetes mortality rates by age. Many efforts have been contributed to developing various glucose sensors [2,3,4,5,6,7,8]. On the other hand, a persistent elevated uric acid (UA) concentration in the blood would result in hyperuricemia. UA is the final metabolic product of purine compounds. Hyperuricemia will lead to gout, hypertension, hypertriglyceridemia, kidney stones and kidney damage, cardiovascular disease, and so on [9]. Moreover, hyperuricemia and insulin resistance share a bidirectional causal effect [10]. The elevated UA concentration in blood is considered one of the best independent predictors of diabetes and commonly precedes the development of both insulin resistance and diabetes type 2 [11]. Therefore, early identification of hyperuricemia could be a promising strategy for preventing diabetes type 2 [12].

The electrochemical sensing platform offers a high-sensitivity, low-cost, compact, and portable assessment tool for metabolic compounds. Recently, there has been much work devoted to glucose and UA simultaneous detection based on electrochemical platforms [13,14,15,16]. However, it is rare of these works to focus on wearable sensing strategy. As we know, the emerging Internet of Things that revolutionizes traditional medical practices presents an excellent opportunity for developing wearable sensing devices [17], which has shown its application potential for continuous monitoring of molecules in forensics, clinics, and healthcare fields [18,19,20,21,22]. Furthermore, the gold standard in human biomarker detection heavily relies on blood analysis, which is an invasive approach. It will inevitably cause pain and psychological pressure on the test subject. Alternatively, developing a non-invasive biomarker monitoring approach can be of great interest, e.g., in sweat matrix. There has been plenty of work in developing sweat-based wearable sensors for monitoring biomarkers [23,24].

**Figure 1:**
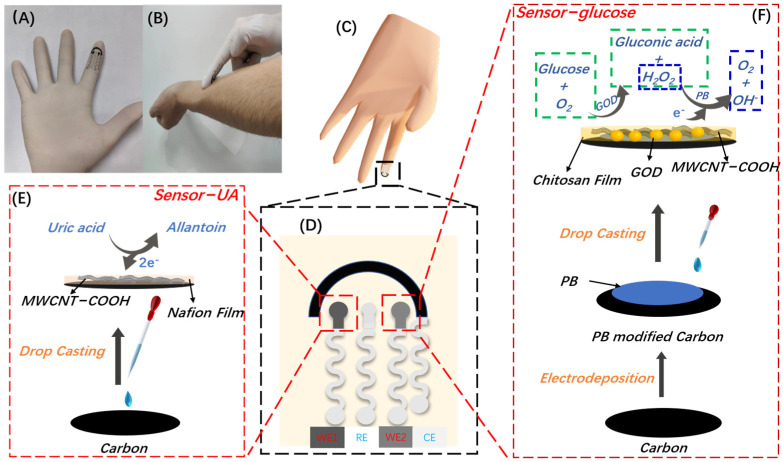
Dual-function electrochemical wearable sensor for glucose and uric acid sensing. (**A**) Optical image of the dual-function wearable electrochemical sensor. (**B**) Optical image of “touch & sense” concept. (**C**) Scheme of the wearable sensor printing on a rubber glove. (**D**) Scheme of the four-electrode sensor. (**E**,**F**) Schematics of the preparations of Sensor-UA and Sensor-glucose and their sensing mechanism towards UA and glucose, respectively.

In this work, we have developed a dual-function wearable electrochemical sensor for uric acid and glucose detection in sweat. The sensor was fabricated on ordinary rubber gloves by printing technique. The proposed four-electrode sensor consists of four serpentine Ag/AgCl wires intended to connect with an electrochemical workstation. The wires’ other ends are printed with carbon or Ag/AgCl ink, which are used as working, counter, and reference electrodes, respectively, as shown in Figure 1A–C. The serpentine design can increase the stretchability of the sensor to a certain extent [25]. The sensors for UA (Sensor-UA) and glucose (Sensor-glucose) were prepared using carboxyl functionalized multiwall carbon nanotubes (MWCNT-COOH) and Prussian blue (PB)-glucose oxidase (GOD)-MWCNT-COOH composite, respectively. The entire sensing process requires only 1 min without finger puncturing. Moreover, the sensor was prepared by printing the electrodes on ordinary rubber gloves, providing a simple and convenient method of sensor preparation. Thus, the proposed dual-function wearable chemical sensor for uric acid and glucose simultaneous detection in sweat can offer a comprehensive monitoring strategy for related disease management and treatment.

## 2. Experimental 

### 2.1. Chemicals and Materials

Na_2_HPO_4_·12H_2_O (≥99%), NaH_2_PO_4_·2H_2_O (≥99%), FeCl_3_ (≥97%), K_3_Fe(CN)_6_ (≥99.5%), KCl (≥99.5%), HCl (36–38%), NaCl(≥99.5%), H_2_O_2_ (30%), acetic acid (≥99.8%), ascorbic acid (≥99.7%), xylene (≥99%), lactic acid (85–92%), NaOH (≥96%), and glucose were purchased from Sinopharm Chemical Reagent Co., Ltd (Shanghai, China). Nafion (5 wt%), chitosan (medium molecular weight), and polystyrene-block-polyisoprene-block-polystyrene (SIS) were obtained from Sigma-Aldrich (Shanghai, China). The carbon and silver/silver chloride (60:40) inks were purchased from SunChemical Co. (Parsippany, NJ, USA). Glucose oxidase (≥100 U/mg) were obtained from Sangon Biotech (Shanghai, China). Uric acid (≥98%) was obtained from TCI Shanghai. –COOH Functionalized Multi-walled Carbon Nanotubes (MWCNT-COOH, ≥98 wt%) was purchased from Chengdu Organic Chemicals Co. Ltd. (Chengdu, China). 0.1 M phosphate-buffered solution (PBS), pH 7.4, was prepared using Na_2_HPO_4_·12H_2_O and NaH_2_PO_4_·2H_2_O. All chemicals were used without further purification. Deionized water from a Milli-Q system (18.2 MΩ·cm at 25 °C) was used throughout the experiments.

### 2.2. Apparatus

The electrochemical assessments were carried out using a CHI-760e workstation (Austin, TX, USA). The electrode printing stencil was cut out on a vinyl transfer film using the Roland desktop cutter GS-24. The morphology was observed using a scanning electron microscope (SEM) ZEISS Gemini 300 (Shanghai, China).

### 2.3. Printing of the Electrodes

To increase the sensor’s stretchability, the ink recipe needed to be adjusted before printing. For the reference electrode and wire-printing ink, 0.4 g Ag/AgCl ink was well-mixed with 0.1 g SIS solution, prepared using 0.5 g SIS dissolving in 1.7 mL xylene. For the working and counter electrodes ink, 0.1 g SIS solution was mixed with 0.3 g carbon ink under the stirring of a magnetic mixer. 

The electrode printing stencil was designed with Adobe Illustrator software (San Jose, CA, USA), as shown in Figure 1D. Then, the stencil was cut out on a vinyl transfer film using the cutter. After the vinyl film was transferred and pasted on rubber gloves, the electrode that was printed out with the ink progressively applied. In detail, the serpentine Ag/AgCl wires with 1.6 mm width were first printed, which were intended to connect with the electrochemical workstation. Then, the Ag/AgCl ink was printed into a disc shape with a 2 mm diameter as the reference electrode. Subsequently, carbon ink was printed as counter and unmodified working electrodes, and each was printed into a disc shape, 3 mm in diameter. At last, the counter electrode was printed as an arc shape with a 1.5 mm width. 

### 2.4. Preparation of UA Sensor (Sensor-UA)

The preparation process has schemed in Figure 1E. First, the working electrode for UA sensing was prepared using MWCNT-COOH-modified carbon electrode. In detail, 2 wt% Nafion was firstly prepared by diluting 5 wt% Nafion stock solution using deionized water. Then, 1 mg MWCNT-COOH was mixed with 1 mL 2 wt% Nafion solution with ultrasound assistance for half an hour. Finally, 3 μL MWCNT-COOH/Nafion composite was drop casted on unmodified carbon working electrode. The prepared electrode, which is marked as working electrode 1 (WE1) in Figure 1D, is referred to as Sensor-UA.

### 2.5. Preparation of Glucose Biosensor (Sensor-Glucose)

The preparation process has schemed in Figure 1F. For the glucose biosensor preparation, the Prussian blue film was electrodeposited on the other unmodified carbon working electrode. The electrodeposition process was similar to our previous work [26]. In detail, the solution containing 2.5 mM FeCl_3_, 2.5 mM K_3_Fe(CN)_6_, 0.1 mM KCl, and 0.1 mM HCl was drop casted on the unmodified carbon working electrode, and then a bias voltage of 0.4 V was applied for 100 s. After rinsing with deionized water, the prepared PB film was activated in a solution of 0.1 mM KCl and 0.1 mM HCl, using cyclic voltammetry from −0.05 V to 0.35 V at the scan rate of 50 mV/s for 15 cycles. Further rinsing with deionized water, the prepared PB-modified electrode was kept in a 75 °C oven for 1 h. This PB-modified working electrode is referred to PB/WE. Secondly, the GOD/MWCNT-COOH/chitosan composite was drop cast on PB/WE. In detail, 2 mg/mL MWCNT-COOH/chitosan solution was prepared by dispersing 2 mg MWCNT-COOH in 1 mL solution containing 1 wt% glacial acetic acid and 2 wt% chitosan. Then, 40 mg/mL GOD was prepared by dissolving GOD in PBS solution. The GOD/MWCNT-COOH/chitosan composite was prepared by mixing GOD solution with MWCNT-COOH/chitosan solution at the volume ratio of 1:2. Finally, 3 μL GOD/MWCNT-COOH/chitosan composite was drop cast on PB/WE, and then kept in 4 °C fridge overnight. The prepared electrode, which is marked as working electrode 2 (WE2) in Figure 1D, is referred to as Sensor-glucose.

### 2.6. Electrochemical Measurements

This study carried the electrochemical measurements in 0.1 M PBS, pH 7.4. Then, 100 μL PBS was drop casted on the electrodes surface for cyclic voltammetry (CV) or chronoamperometry experiments. The corresponding chemicals for detection were spiked into the PBS to achieve the desired concentration. For a real sample test, the sweat was collected from a volunteer after exercise. Then, 100 μL collected sweat was used as an electrolyte by drop casting on the sensors without further treatment. Similarly, the chemicals for test were spiked into the sweat. Chronoamperometry was carried out by applying the corresponding potential on the working electrodes for 60 s. The current response was recorded by reading the current value at 60 s. 

## 3. Results and Discussion

### 3.1. Sem Characterization

**Figure 2:**
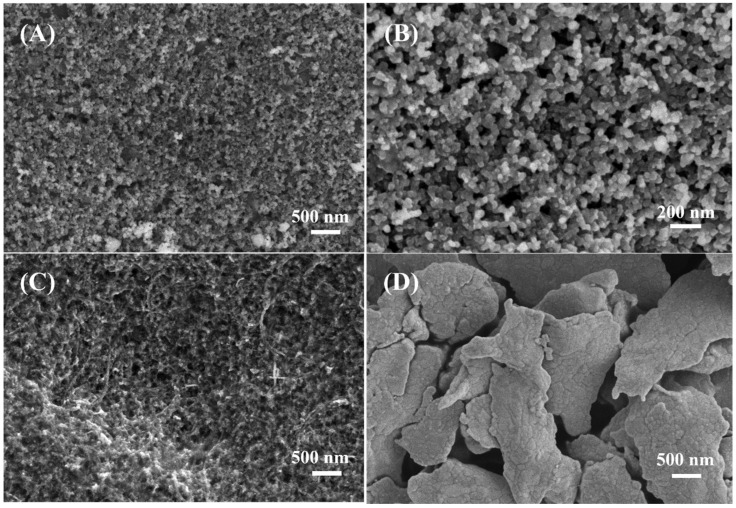
SEM images of (**A**,**B**) unmodified bare carbon electrode with different magnifications, (**C**) MWCNT-COOH modified carbon electrode and (**D**) Ag/AgCl electrode.

Figure 2 show the SEM morphologies of the unmodified bare carbon electrode, MWCNT-COOH modified carbon electrode and Ag/AgCl electrode, respectively. As Figure 2A,B exhibit, unmodified bare carbon electrode is composed of nano-carbon balls tightly stacking together, which allows the smooth flow of electrons and shows good conductivity of the electrode. Figure 2C is the SEM image of MWCNT-COOH modified carbon electrode, and MWCNT-COOH is evenly distributed on the electrode surface. Figure 2D is the SEM image of Ag/AgCl electrode. We can find that the morphology of Ag/AgCl is flaky shape with layers stacking, which constructs the electron pathways.

### 3.2. Electrochemical Characterization

**Figure 3:**
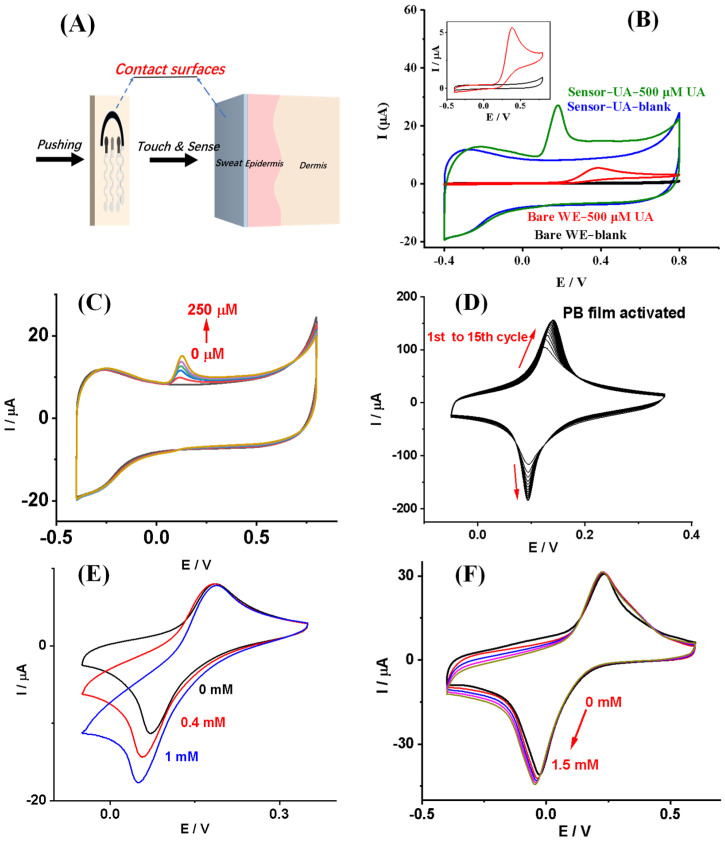
Electrochemical performance of the sensor. (**A**) Scheme of the working process of “Touch & Sense”. (**B**) CVs of the electrodes in PBS (blank) and PBS containing 500 μM UA. The inset shows the CVs of unmodified carbon working electrode (bare WE) to blank and 500 μM UA in PBS, respectively. Scan rate 50 mV/s, potential range −0.4 V to 0.8 V. (**C**) CVs of Sensor-UA towards 0, 45, 100, 150, 200 and 250 μM UA in PBS, respectively. Scan rate 50 mV/s, potential range −0.4 V to 0.8 V. (**D**) CVs of PB film in 0.1 mM KCl and 0.1 mM HCl solution for activation. Scan rate 50 mV/s, potential range −0.05 V to 0.35 V, 15 cycles. (**E**) CVs of PB modified carbon working electrode towards 0.1 M PBS + 0.1 M KCl containing 0, 0.4 and 1 mM H_2_O_2_, respectively. Scan rate 50 mV/s, potential range −0.05 V to 0.35 V. (**F**) CVs of Sensor-glucose towards 0, 0.4, 0.8, 1.2, and 1.5 mM glucose in 0.1 M PBS, respectively. Scan rate 50 mV/s, potential range −0.4 V to 0.6 V.

The prepared electrochemical wear sensor provides a fast and convenient sensing strategy for simultaneous uric acid and glucose sensing based on the “Touch & Sense” working concept, as shown in Figure 3A. To characterize the sensor’s electrochemical performance, cyclic voltammetry was carried out. Figure 3B shows CVs of the electrodes, unmodified bare carbon electrode or MWCNT-COOH modified carbon electrode (Sensor-UA), in PBS (blank) or PBS containing 500 μM UA. As we can see, after 500 μM UA was added to the electrodes, unmodified bare carbon electrode or Sensor-UA, an anodic peak appeared. This should be due to the electrochemical oxidation of UA on the electrodes. The electrochemical oxidation mechanism is shown in Figure 1E. In detail, uric acid was oxidized on MWCNT-COOH modified carbon electrode with two electrons transferred, giving the product of allantoin. It indicates that, after the modification of MWCNT-COOH, the electrodes show an increased electrical double-layer capacitance. It can be ascribed to the increased electrode area brought from MWCNT-COOH. 

Moreover, UA’s electrochemical oxidation peak potentials were found at 0.38 V for unmodified bare carbon electrodes while at 0.18 V for MWCNT-COOH modified carbon electrodes. In the meantime, as we can see from Figure 3B’s inset, the onset oxidation potential of UA on bared carbon electrode is around 0.16 V (vs. Ag/AgCl), while for Sensor-UA is around 0.06 V (vs. Ag/AgCl). The reduction of UA electrochemical oxidation peak and onset potentials was ascribed to the modification of MWCNT-COOH on bare carbon electrodes. Thus, the modification of MWCNT-COOH on carbon not only increases the electrode’ area, but also reduces the over oxidation potential of UA on the electrode. This indicates the improved electrochemical catalytic oxidation performance of MWCNT-COOH towards UA [27]. Figure 3C shows the CVs of Sensor-UA towards 0, 45, 100, 150, 200, and 250 μM UA in PBS, respectively. As the UA concentration increased, the oxidation peak current of UA increased. The Sensor-UA has exhibited its potential sensing application towards UA. Figure 3D shows the CVs of PB film in 0.1 mM KCl and 0.1 mM HCl solution for activation. As the scanning processed, the well-defined redox peaks of PB increased. As we expected, PB has been modified on the carbon electrode. Figure 3E shows the CVs of PB-modified carbon working electrode towards 0.1 M PBS and 0.1 M KCl containing 0, 0.4 and 1 mM H_2_O_2_, respectively. As the H_2_O_2_ concentration increased, the PB-modified electrode’s reduction peak current increased. We have discussed the PB-modified electrodes’ electrochemical reaction mechanism towards H_2_O_2_ in our previous work [26]. For glucose sensing mechanism, glucose is enzymatically oxidized by glucose oxidase in presence of oxygen, giving one of the products H_2_O_2_. Then, H_2_O_2_ is electrochemically reduced on PB modified electrode. Figure 3F shows CVs of Sensor-glucose towards 0, 0.4, 0.8, 1.2, and 1.5 mM glucose in 0.1 M PBS, respectively. As we have discussed, MWCNT-COOH can increase the electrode’s area. So, the redox current of Sensor-glucose is more significant than that of the PB-modified electrode after GOD/MWCNT-COOH/chitosan composite’s modification. The electrochemical reaction mechanism of H_2_O_2_ and glucose on Sensor-glucose is shown in Figure 1F. As glucose concentration increased, the reduction peak current of Sensor-glucose increased, which indicates the Sensor-glucose’s potential sensing application towards glucose.

### 3.3. Electrochemical Sensing Performance

**Figure 4:**
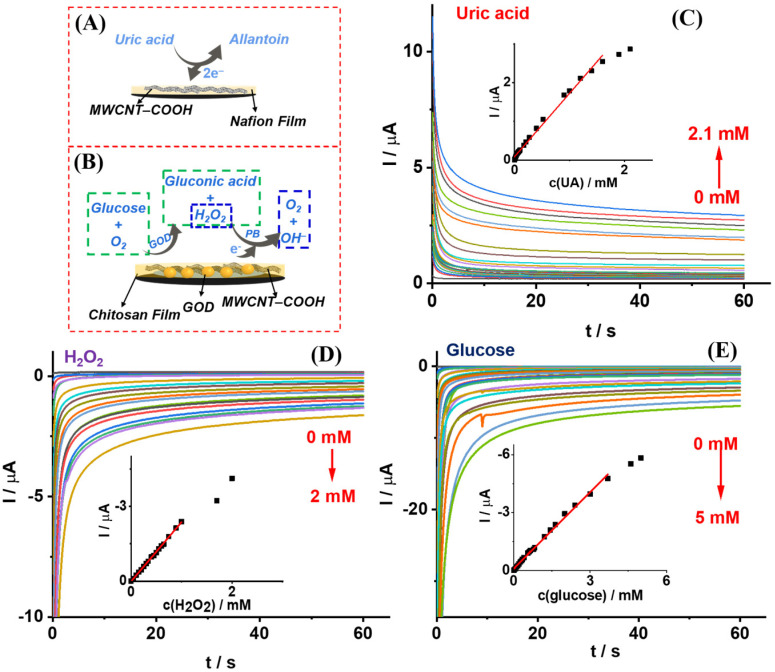
The electrochemical sensing performance of the dual function sensor in 0.1 M PBS, pH 7.4. (**A**,**B**) Schemes of Sensor-UA and Sensor-glucose’s electrochemical reaction mechanism towards UA and glucose, respectively. (**C**–**E**) Chronoamperometric response of Sensor-UA, PB/WE and Sensor-glucose towards UA, H_2_O_2_ and glucose, respectively. The insets show the corresponding calibration curves with linear fitting, *n* = 3. The applied potentials were 0.12, 0.03 and 0.03 V, respectively. All of the recording times were 60 s.

To evaluate the dual-sensor’s electrochemical sensing performance, chronoamperometric experiments were carried out in 0.1 M PBS, pH 7.4. Figure 4A,B show the electrochemical reaction mechanism of Sensor-UA and Sensor-glucose towards UA and glucose, respectively. For Sensor-UA’s sensing evaluation, a potential of 0.12 V was applied for 60 s to record the current response. As shown in Figure 4C, UA’s electrochemical oxidation current increased as UA concentrations increased. The inset shows the calibration curve obtained from the chronoamperometric response. The linear fitting curve falling within the UA concentration range from 0 to 1.6 mM follows the equation:

I (μA) = 1.638 × c(UA) (mM) + 0.0845 (R^2^ = 0.9936)

The limit of detection (LOD) of 3.58 μM was obtained for Sensor-UA (S/N = 3) in PBS.

To evaluate the sensing performance of Sensor-glucose, the PB/WE’s sensing performance towards H_2_O_2_ was first investigated using chronoamperometry at a constant potential of 0.03 V for 60 s. As shown in Figure 4D, as H_2_O_2_ concentrations raised from 0 to 2 mM, the electrochemical reduction current increased. The inset has plotted the linear fitting curve from the chronoamperometric response within H_2_O_2_ concentrations ranging from 0 to 1 mM. The curve follows the equation:

I (μA) = −2.379 × c(H_2_O_2_) (mM) + 0.0079 (R^2^ = 0.9990)

Additionally, the LOD of PB/WE towards H_2_O_2_ was calculated as 9.45 μM (S/N = 3) in PBS.

After the GOD/MWCNT-COOH/chitosan composite was modified on PB/WE, Sensor-glucose was prepared. The sensing performance of Sensor-glucose towards glucose was investigated with a constant potential of 0.03 V applied for 60 s. As we can see from Figure 4E, the electrochemical reduction current of the sensor increased as glucose concentration increased from 0 to 5 mM. The inset has shown the calibration curve of the Sensor-glucose towards glucose. The linear fitting curve was obtained within the glucose concentration from 0 to 3.7 mM. The fitting curve’s equation follows:

I (μA) = −1.322 × c(glucose) (mM) − 0.0970, R^2^ = 0.9950

Additionally, the LOD of Sensor-glucose towards glucose was calculated as 9.10 μM (S/N = 3) in PBS.

### 3.4. Stability, Selectivity, and Real Sample Test

**Figure 5:**
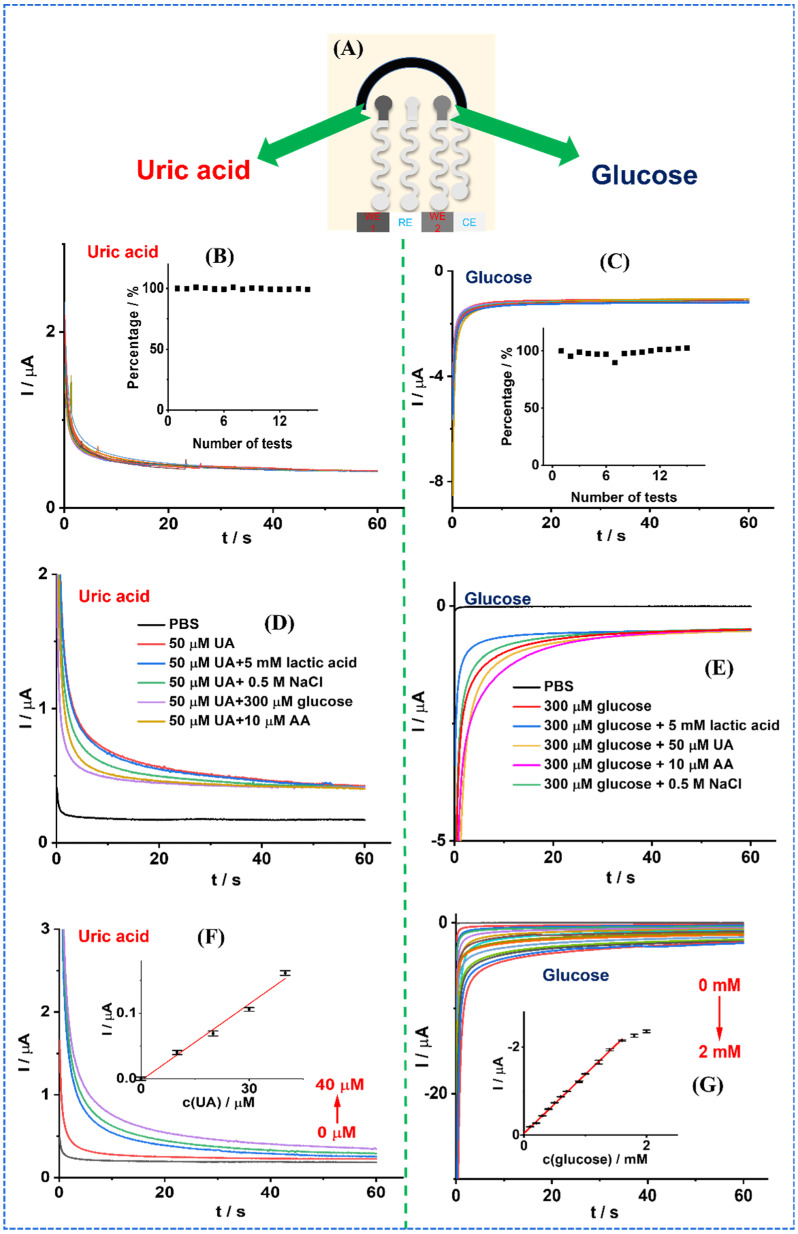
The stability, selectivity, and real sample sensing of the dual-function wearable electrochemical sensor. (**A**) Schematic of the sensor’s design. (**B**,**C**) The stability of the sensor for UA and glucose sensing by chronoamperometry for 60 s, respectively. The insets plot the degree of response attenuation of 15 tests compared to the 1st chronoamperometric response. (**D**,**E**) The selectively test of the dual sensor for UA and glucose sensing, respectively. The common interfering species and their corresponding concentrations in physiological conditions are listed in the Figure. (**F**,**G**) The dual function sensor’s real sample test for UA and glucose in sweat, respectively. The insets show the relationship of response current against the concentration, and the linear fitting curves are plotted, respectively. *n* = 3.

The chronoamperometry was performed to assess the electrochemical sensor’s stability. The sensor’s design is illustrated in Figure 5A. The stability test for Sensor-UA is shown in Figure 5B. A potential of 0.12 V was applied for 60 s to record the current response. As we can see, during 15 tests, the current response almost kept the same. The inset shows the degree of response attenuation after 15 tests, and the attenuation is virtually negligible, indicating the excellent stability of Sensor-UA. Figure 5C illustrates the stability test of Sensor-glucose. A potential of 0.03 V was applied for 60 s. Similarly, the inset shows the degree of response attenuation of 15 tests compared to the 1st chronoamperometric response. Again, the attenuation of the response is almost negligible, indicating the good stability of Sensor-glucose.

Selectively is one of the crucial evaluation indicators for sensors. The chronoamperometry was conducted for 60 s to assess the selectively, with a constant potential applied. The common interfering species at their respective physiological concentration levels were spiked onto the sensors, e.g., 50 μM UA (for glucose sensing), 300 μM glucose (for UA sensing), 5 mM lactic acid, 0.5 M NaCl, and 10 μM ascorbic acid (AA). As shown in Figure 5D,E, the sensors showed their specific response towards their targets, UA and glucose. However, with the addition of interfering species, the current response was negligible. This indicates that the dual-function sensor has shown good detection selectively.

To assess the feasibility of the sensors for real sample sensing, fresh sweat was collected from a volunteer after exercise. First, 100 μL of the sweat was drop cast on the sensors, then different concentrations of UA (0, 10, 20, 30, and 40 μM) and glucose (0, 0.1, 0.2, 0.3, 0.4, 0.5, 0.6, 0.7, 0.9, 1.0, 1.22, 1.4, 1.6, 1.8 and 2.0 mM) were subsequently spiked onto the electrode surface, respectively. As shown in Figure 5F, as the spiked UA concentrations increased from 0 to 40 μM, the chronoamperometric response of Sensor-UA increased. The inset shows the linear fitting curve obtained from the chronoamperometric response of Sensor-UA to UA. The relationship of response and spiked UA concentration in real sample follows this equation:

I (μA) = 0.00389 × c(UA) (μM) − 0.00246, R^2^ = 0.9870

For glucose sensing in sweat, as shown in Figure 5G, as the spiked glucose concentrations increased from 0 to 2 mM, the chronoamperometric reduction current of Sensor-glucose increased. The inset shows the linear fitting curve obtained from the chronoamperometric response of Sensor-glucose to glucose within the glucose concentration range from 0 to 1.6 mM in sweat. The fitting curve’s equation is:

I (μA) = −1.341 × c(glucose) (mM) − 0.0401, R^2^ = 0.9985

It is worth mentioning that the linear detection range of Sensor-UA (0 to 40 μM) and Sensor-glucose (0 to 1.6 mM) for extra added UA and glucose can cover the concentration of UA (24.5 to 35.7 μM) [28] and glucose (0.01 to 1.11 mM) [29] in sweat under physiological conditions. This further indicates the feasibility of this dual-function sensor in real sample detection.

## 4. Conclusions

In this study, we have proposed a dual-function wearable electrochemical sensor for simultaneous uric acid and glucose sensing in sweat. The sensor was printed on an ordinary rubber glove with two modified working electrodes for uric acid and glucose sensing, respectively. The specific sensing performance of the sensor for disease-related molecules are based on electrochemical reaction mechanism. Uric acid is directly oxidized on MWCNT-COOH modified carbon electrode, and glucose is enzymatically oxidized on glucose oxidase biofilm then the obtained product H_2_O_2_ is electrochemically reduced on Prussian blue modified electrode. Moreover, the sensors exhibited a fast, high sensitivity and selectivity sensing performance. The proposed sensors have been tested and demonstrated their feasibility in sweat sensing, and the sensing range well covers the concentration range of UA and glucose in physiological conditions. The proposed dual-function wearable chemical sensor can provide a noninvasive, comprehensive monitoring strategy for uric acid and glucose-related disease management and treatment.

## Data Availability

Data sharing is not applicable to this article.
